# Method Adaptation Patterns and a Selection Framework for Near-Infrared Spectroscopy-Based Moisture Prediction of Major Grain Crops

**DOI:** 10.3390/foods15173093

**Published:** 2026-08-31

**Authors:** Chenxiao Li, Sheng Wang, Qinglong Zhao, Pei Wang, Qian Song, Daping Fu

**Affiliations:** 1 College of Information Technology, Jilin Agricultural University, Changchun 130118, China; licx@jlau.edu.cn (C.L.); wangsheng@mails.jlau.edu.cn (S.W.); zhaoqinglong@mails.jlau.edu.cn (Q.Z.); wpei2280@163.com (P.W.); 2 College of Physics, Jilin University, Changchun 130012, China; songqian@jlu.edu.cn; 3 College of Engineering and Technology, Jilin Agricultural University, Changchun 130118, China

**Keywords:** moisture content, near-infrared spectroscopy, preprocessing, feature selection, prediction model, nondestructive testing, scenario adaptation

## Abstract

Moisture content is an important indicator of grain storage safety, processing quality, and circulation efficiency. However, the performance of near-infrared spectroscopy (NIRS) models is affected by differences in grain crops, sample morphologies, preprocessing strategies, feature selection methods, and regression models, and the adaptation relationships among these factors remain insufficiently understood. In this study, soybean, maize, and wheat samples with whole-grain and powder morphologies were investigated to reveal the method adaptation patterns of NIRS-based moisture prediction under consistent experimental conditions. Spectra in the range of 900–1700 nm were collected, and 54 analytical pathways were established by combining six preprocessing strategies, three feature selection algorithms (SPA, CARS, and UVE), and three regression models (PLSR, SVR, and RF). The results showed that representative optimal pathways achieved Rp values above 0.9730 for powder samples and above 0.9587 for whole-grain samples. Different grain crops and sample morphologies exhibited distinct analytical pathway preferences, indicating that appropriate analytical strategies should be selected according to specific detection objects. Spectral variation analysis further demonstrated higher spectral variability in whole-grain samples than in powder samples. This study provides insights into the selection of suitable NIRS analytical strategies for grain moisture prediction and quality assessment.

## 1. Introduction

Soybean, maize, and wheat are among the most important grain crops worldwide and play essential roles in global food production, trade, and food security. Moisture content (MC) is a critical quality parameter that influences grain storage safety, processing performance, transportation, and quality preservation. Excessive moisture content can promote microbial growth, mycotoxin contamination, grain aggregation, and nutrient degradation, whereas insufficient moisture may increase kernel breakage, reduce seed viability, and negatively affect processing quality. Therefore, accurate and rapid determination of grain moisture content is essential for storage management, quality evaluation, and processing applications [1].

The oven-drying method remains the internationally recognized reference method for grain moisture determination. However, this method is destructive, time-consuming, and labor-intensive, usually requiring several hours for a single measurement. Therefore, it is difficult to meet the increasing demand for rapid, nondestructive, and high-throughput analysis in modern grain industries. Developing rapid, accurate, and nondestructive moisture detection technologies has therefore become an important research direction in agricultural quality control.

In addition to conventional reference methods, several instrumental techniques have been developed for rapid grain moisture determination, including resistance-, capacitance-, radiation-, microwave-, and near-infrared-based methods. Resistance- and capacitance-based methods are relatively inexpensive and provide rapid measurements, but their accuracy can be affected by factors such as material distribution, bulk density, contact conditions, and temperature. Radiation-based methods offer rapid, nondestructive measurement and good penetration capability; however, radiation safety concerns and relatively high equipment costs limit their routine application in agricultural testing. Microwave-based methods provide rapid, nondestructive, and non-contact measurement with relatively strong penetration, but their measurement performance can be influenced by grain density, material composition, standing-wave or multipath effects, and characteristics of the measurement system, often requiring appropriate calibration or compensation strategies [2,3]. NIRS also enables rapid and nondestructive moisture determination with minimal sample preparation, although its measurement performance may be affected by physical characteristics of the sample, such as particle size and density. Importantly, NIR absorption arises from overtones and combination bands of fundamental molecular vibrations, with X–H bonds such as O–H, C–H, and N–H providing important absorption features in the near-infrared region. For moisture determination, O–H-related absorption provides direct spectral information associated with water content [4]. Combined with chemometric methods, NIRS can establish quantitative relationships between these spectral responses and moisture content. Its rapidity, nondestructive nature, minimal sample preparation requirements, and compatibility with multivariate analytical methods make NIRS particularly suitable for the systematic comparison of preprocessing, feature-selection, and modeling strategies investigated in this study.

Compared with conventional oven-drying methods, NIRS also offers the potential for simultaneous assessment of multiple quality attributes, online monitoring, and large-scale inspection. It has therefore been widely applied in grain quality evaluation and rapid agricultural product analysis [5]. With the continuous development of chemometrics and machine learning techniques, NIRS-based grain moisture prediction has been further advanced, making it an important tool for modern grain quality assessment.

In recent years, NIRS has been extensively applied in agricultural product quality evaluation. Numerous studies have demonstrated that the combined optimization of spectral preprocessing, feature wavelength selection, and chemometric modeling is essential for improving prediction accuracy and model robustness [6]. For major grain crops such as soybean, maize, and wheat, previous studies have confirmed the feasibility of NIRS and hyperspectral imaging technologies for nondestructive moisture and quality assessment. Various spectral techniques, including NIRS, long-wave near-infrared spectroscopy, and visible–near-infrared hyperspectral imaging, have been successfully applied to moisture prediction, quality evaluation, and dynamic monitoring of drying processes in maize, rice, glutinous rice, oats, and other cereal crops [7,8,9,10,11]. These studies have provided important theoretical foundations and technical support for nondestructive grain moisture detection.

Recent reviews have systematically summarized the development of NIRS applications in grain quality assessment. Liang et al. [12] reviewed the application of hyperspectral imaging in grain quality and safety evaluation, highlighting that the combined optimization of spectral preprocessing, feature variable selection, and machine learning models can significantly improve moisture prediction accuracy and model generalization ability. Ageh et al. [13] summarized the applications of various optical imaging techniques, including near-infrared hyperspectral imaging, in grain quality evaluation and discussed the major challenges and future prospects associated with the transition from laboratory-based analysis to industrial online detection. These studies demonstrate the considerable potential of NIRS and hyperspectral imaging for nondestructive grain moisture analysis and provide important methodological foundations for further development.

To improve the predictive performance and practical applicability of NIRS models, extensive efforts have been devoted to model generalization, analytical workflow optimization, and sample adaptability. To address the limited generalization ability of prediction models, Zheng et al. [14] integrated gradient boosting algorithms into the feature selection process of partial least squares regression and developed a universal model for predicting moisture and protein contents in maize kernels from multiple production regions. Wang et al. [15] combined a portable NIRS instrument with machine learning algorithms to achieve rapid nondestructive detection of wheat powder gluten quality. Xu et al. [16] further emphasized that diverse sample sets covering different origins and varieties are essential for improving model robustness. However, most existing studies remain focused on individual grain varieties.

Regarding analytical workflow optimization, Zhang et al. [17] systematically compared the effects of spectral preprocessing, feature selection, and modeling methods for wheat powder quality evaluation, although only powder samples were considered. Meanwhile, Yan et al. [18], Möhring et al. [19], and Mishra et al. [20] reviewed the development of NIRS preprocessing strategies from the perspectives of preprocessing principles, combined preprocessing optimization, and integrated preprocessing approaches, providing valuable references for systematic comparison of different preprocessing methods. Recently, increasing attention has been given to the integration of feature selection algorithms and machine learning models for quantitative quality analysis and variety identification of cereal crops such as rice and wheat [21,22,23,24]. Geng et al. [25] further investigated NIRS modeling under different sample morphologies and improved model adaptability to different sample states. However, most previous studies have focused on a single grain variety, a single sample morphology, or a limited moisture range. A systematic evaluation integrating different grain varieties, sample morphologies, and complete analytical workflows is still lacking.

To address these limitations, this study investigated soybean, maize, and wheat as representative major grain crops and established a comparable sample system under unified experimental conditions. Both whole-grain and powder samples were prepared, and a broad moisture range of approximately 5–30% was designed to represent moisture variations encountered during grain storage, transportation, and processing. By combining six spectral preprocessing strategies, three feature selection algorithms, and three regression models, 54 analytical pathways were constructed and systematically evaluated for different grain varieties and sample morphologies. Based on these comparisons, the method adaptation patterns of NIRS-based moisture prediction were further summarized.

The major contributions of this study are as follows: (1) A parallel evaluation system covering soybean, maize, and wheat was established to systematically investigate differences in NIRS moisture prediction strategies among major grain crops under consistent experimental conditions, extending previous studies mainly focused on individual crops. (2) Both whole-grain and powder sample morphologies were considered within the same evaluation framework, enabling analysis of the influence of sample morphology on spectral characteristics, feature extraction, and model construction strategies. (3) A comprehensive evaluation system consisting of 54 analytical pathways was developed by integrating preprocessing, feature selection, and regression modeling. Furthermore, the method adaptation patterns between different detection objects and analytical strategies were summarized, and a NIRS-based method selection framework for grain moisture prediction was proposed.

Rather than pursuing a universal optimal prediction model, this study aims to reveal the relationships between different detection objects and analytical strategies. The proposed framework provides guidance for selecting appropriate NIRS analytical pathways under different grain varieties, sample morphologies, and application scenarios, and offers a reference for future model transfer, external validation, and rapid grain quality assessment.

## 2. Materials and Methods

### 2.1. Sample Preparation

The experimental materials consisted of three major grain crops: soybean, maize, and wheat. To improve sample representativeness, samples from different batches were collected and thoroughly mixed before experiments to reduce the influence of source-related variations on spectral characteristics. Prior to preparation, all samples were cleaned and manually screened, and only intact grains with normal color and without visible mechanical damage or mold contamination were selected to ensure sample consistency and comparability.

To simulate the variations in moisture conditions that may occur during grain harvesting, storage, and processing, 13 moisture levels were prepared for each mixed sample. The moisture ranges were 6.65–26.87% for soybean, 9.64–29.32% for maize, and 9.38–25.72% for wheat. Initially, approximately 5 g of randomly selected samples were ground and analyzed using the oven-drying method to determine the initial moisture content. The wet weight (d_1_) was measured using an electronic balance with a precision of 0.001 g. The samples were then dried in an oven at 110 °C for 3 h and cooled to room temperature before measuring the dry weight (d_2_). The initial moisture content was calculated according to Equation (1):

(1) $$y\%=\frac{\left(d_{1}-d_{2}\right)}{d_{1}}\times 100\%$$

Based on the target moisture levels, the required amount of distilled water was calculated for each gradient. The calculated water volume was uniformly sprayed onto the sample surface, followed by thorough mixing. The samples were then sealed and equilibrated at 4 °C for 1–2 days to allow sufficient water diffusion and achieve uniform moisture distribution. After equilibration, the samples were returned to room temperature, and the actual moisture content of each gradient was remeasured using the oven-drying method to verify the accuracy of moisture preparation.

After moisture gradient preparation, each sample was divided into two groups. One group was maintained in the whole-grain state for nondestructive spectral measurement, whereas the other group was ground for approximately 30 s using a grinder to obtain homogeneous powder samples for comparative analysis. Sample preparation and spectral measurements were conducted under laboratory conditions, with the ambient temperature ranging approximately from 23 to 26 °C and the relative humidity from 45% to 60% during the experimental period. Spectral acquisition was performed within a similar daytime period to reduce temporal variation in the measurement environment. After preparation, all samples were immediately sealed to minimize moisture exchange with the surrounding environment and were opened only when required for spectral measurement.

### 2.2. Spectral Measurement

**Figure 1:**
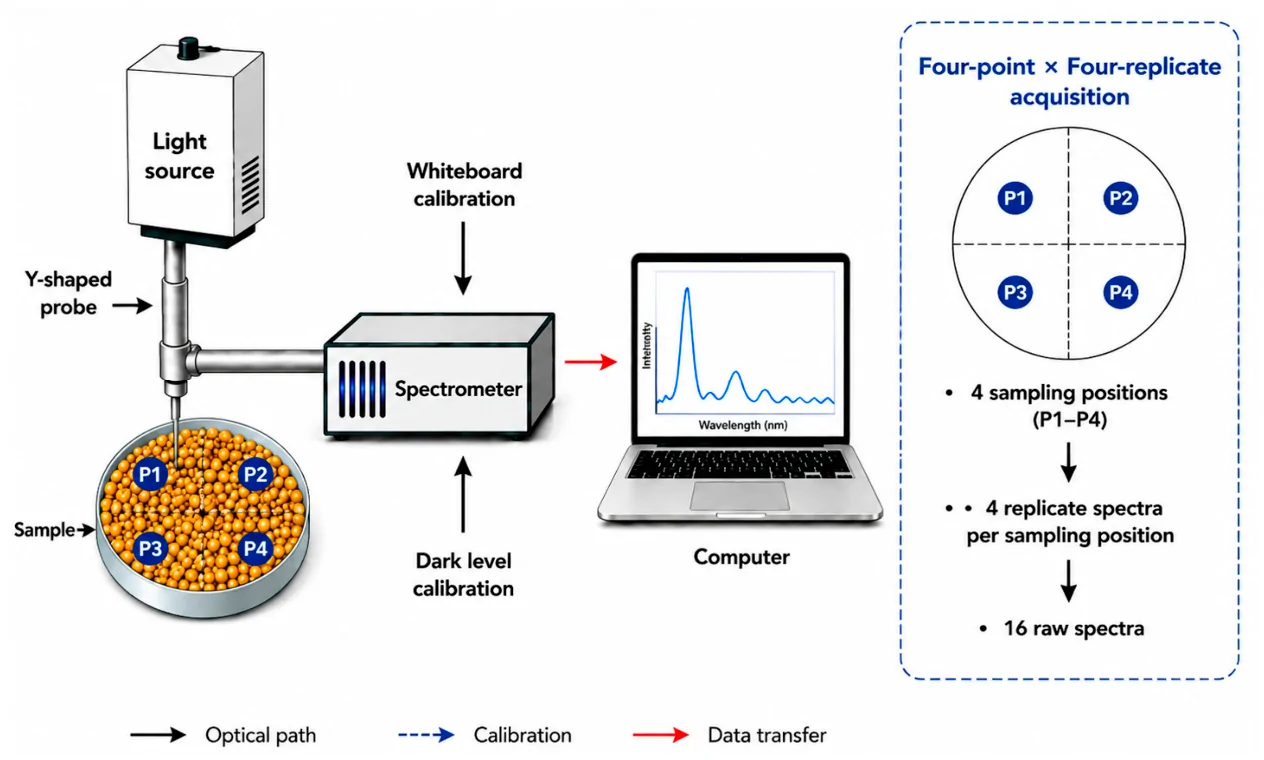
Schematic illustration of the near-infrared spectral acquisition system and the four-position × four-acquisition sampling strategy.

Near-infrared spectra were collected under the laboratory conditions described in Section 2.1 for both whole-grain and powder samples of soybean, maize, and wheat. The measurements were performed using an Ocean Insight NIRQuest512-2.5 near-infrared spectrometer equipped with an OSRAM HLX64625 halogen tungsten lamp as the light source. The configuration of the NIR spectral acquisition system and the “four positions × four separate spectral acquisitions” sampling strategy are illustrated in Figure 1.

The near-infrared radiation emitted from the light source was directed onto the sample surface, and the diffuse reflected light was transmitted to the spectrometer through a Y-shaped optical fiber for spectral acquisition. The spectra were collected within the wavelength range of 900–1700 nm, with an optical resolution of approximately 10 nm and a signal-to-noise ratio (SNR) higher than 1000:1. All samples were placed in clean and dry quartz sample cups during measurement to minimize external interference and ensure the stability of spectral signals.

To improve the representativeness of spectral data and reduce the influence of sample spatial heterogeneity on measurement results, a “four positions × four separate spectral acquisitions” strategy was applied to each moisture-gradient sample. Specifically, the sample surface was divided into four measurement areas (P1–P4), and four separate spectral acquisitions were performed at each position. For each acquisition, the spectral signal was refreshed and newly recorded by OceanView software (version 2.0) such that each measurement produced a separately acquired spectrum rather than a duplicated spectral record. Thus, 16 spectra (four positions × four acquisitions) were obtained for each moisture level. Since 13 moisture levels were prepared for each grain type, a total of 13 × 16 = 208 raw spectra were obtained for each sample morphology.

Before each measurement, white reference and dark current calibrations were performed to ensure the stability and accuracy of spectral acquisition. The sample reflectance was calculated according to Equation (2):

(2) $$R=\frac{I_{s}-I_{d}}{I_{w}-I_{d}}$$

where $I_{s}$, $I_{d}$, and $I_{w}$ represent the reflected intensity of the sample, the dark current signal, and the white reference intensity, respectively. The calculated reflectance spectra were imported into the Python 3.7 environment for subsequent spectral preprocessing, feature selection, and regression model development.

Finally, near-infrared spectral datasets were obtained for six sample categories, including soybean whole-grain, soybean powder, maize whole-grain, maize powder, wheat whole-grain, and wheat powder samples. Each category contained 208 raw spectra, resulting in a total of 1248 raw spectra.

### 2.3. Data Processing and Modeling

Raw near-infrared spectra usually contain non-target information, including background noise, baseline drift, and scattering effects, which may reduce the prediction accuracy and stability of calibration models. Therefore, spectral preprocessing is required before model development to minimize the influence of irrelevant variations and enhance the correlation between spectral features and moisture content.

To systematically evaluate the effects of different preprocessing methods on model performance, spectral data within the effective wavelength range of 900–1700 nm were preprocessed using Python. The preprocessing methods included Savitzky–Golay (SG) smoothing, first derivative of SG (SGD1), second derivative of SG (SGD2), multiplicative scatter correction (MSC), and standard normal variate (SNV) transformation. Based on these preprocessing methods, six combined preprocessing strategies were further established, including SG + D1, SG + D2, MSC + D1, MSC + D2, SNV + D1, and SNV + D2.

These preprocessing strategies were subsequently combined with three feature selection algorithms (SPA, CARS, and UVE) and three regression models (PLSR, SVR, and RF), resulting in 54 analytical pathways. These pathways were systematically evaluated to investigate the effects of different analytical workflows on grain moisture prediction performance.

All preprocessing procedures were performed in the Python 3.7 environment, and the processed spectral datasets were further used for feature selection and regression model development.

### 2.4. Feature Selection, Regression Modeling, and Analytical Workflow

#### 2.4.1. Feature Selection

Near-infrared (NIR) spectral data usually contain a large number of variables, redundant wavelength information, and strong collinearity among variables. To improve model prediction performance, reduce computational complexity, and select characteristic wavelengths highly related to moisture content, feature selection was performed on the preprocessed spectra within the range of 900–1700 nm. Three classical feature selection methods, including the Successive Projections Algorithm (SPA), Competitive Adaptive Reweighted Sampling (CARS), and Uninformative Variable Elimination (UVE), were compared in this study.

With the development of wavelength selection methods for NIR spectroscopy, Ma et al. [26] proposed a wavelength selection method based on maximum relevance minimum redundancy. This method improves feature selection efficiency by considering both the correlation between variables and the target property and the redundancy among variables, providing a new strategy for NIR spectral feature selection. Considering that SPA, CARS, and UVE have been widely applied in NIR-based quantitative analysis of agricultural products, these three classical methods were selected for systematic comparison in this study.

SPA reduces variable collinearity by sequentially selecting wavelength variables with the maximum projection values. The maximum number of selected features was set to 30, and cross-validation was applied to determine the optimal feature subset. CARS performs wavelength selection based on Monte Carlo sampling and regression coefficients from partial least squares regression (PLSR). In this study, a total of 50 Monte Carlo sampling iterations were performed. During the iterative process, wavelength variables with relatively low contributions were progressively eliminated, and the optimal iteration was determined according to the cross-validation error to obtain the final characteristic wavelength subset. The Monte Carlo procedure was used specifically for CARS-based variable selection rather than for repeated training–testing validation of the regression models. UVE introduces random noise variables to calculate the stability index of each wavelength, removes uninformative variables, and retains effective wavelengths related to moisture content.

After spectral preprocessing, SPA, CARS, and UVE were separately applied to spectral data under different preprocessing conditions for wavelength selection. This process was used to evaluate the compatibility between preprocessing strategies and feature selection methods and to provide effective spectral variables for subsequent regression modeling.

#### 2.4.2. Regression Modeling

Based on the selected characteristic wavelengths, three regression models, including partial least squares regression, support vector regression, and random forest, were established to predict grain moisture content and compare their prediction performance.

For PLSR, the optimal number of latent variables was determined using 5-fold cross-validation within the training set, with the minimum root mean square error of cross-validation used as the optimization criterion. SVR employed a radial basis function (RBF) kernel, and the penalty parameter C and kernel parameter γ were optimized using grid search combined with 5-fold cross-validation within the training set. RF was developed as an ensemble regression model through parameter optimization to improve prediction stability.

All models used the selected characteristic variables as input, and the spectral datasets were divided into training and testing sets at a ratio of 7:3. The data partition was performed at the moisture-level group level, such that the repeated spectral observations obtained from the same moisture level were assigned to the same subset and were not simultaneously included in both the training and testing sets. This strategy was used to reduce the risk of information leakage arising from highly correlated repeated spectral observations. The same data-partitioning strategy was consistently applied across all analytical pathways to ensure a fair and comparable evaluation among different preprocessing methods, feature selection algorithms, and regression models.

By combining six preprocessing strategies, three feature selection methods, and three regression models, a total of 54 near-infrared analytical pathways (54 analytical pathways) were constructed. These analytical pathways were applied to three grain species (soybean, maize, and wheat) and two sample morphologies (powder and whole-grain) to systematically evaluate the prediction performance of different analytical strategies and further investigate the adaptation patterns among spectral preprocessing methods, feature selection algorithms, and regression models.

**Figure 2:**
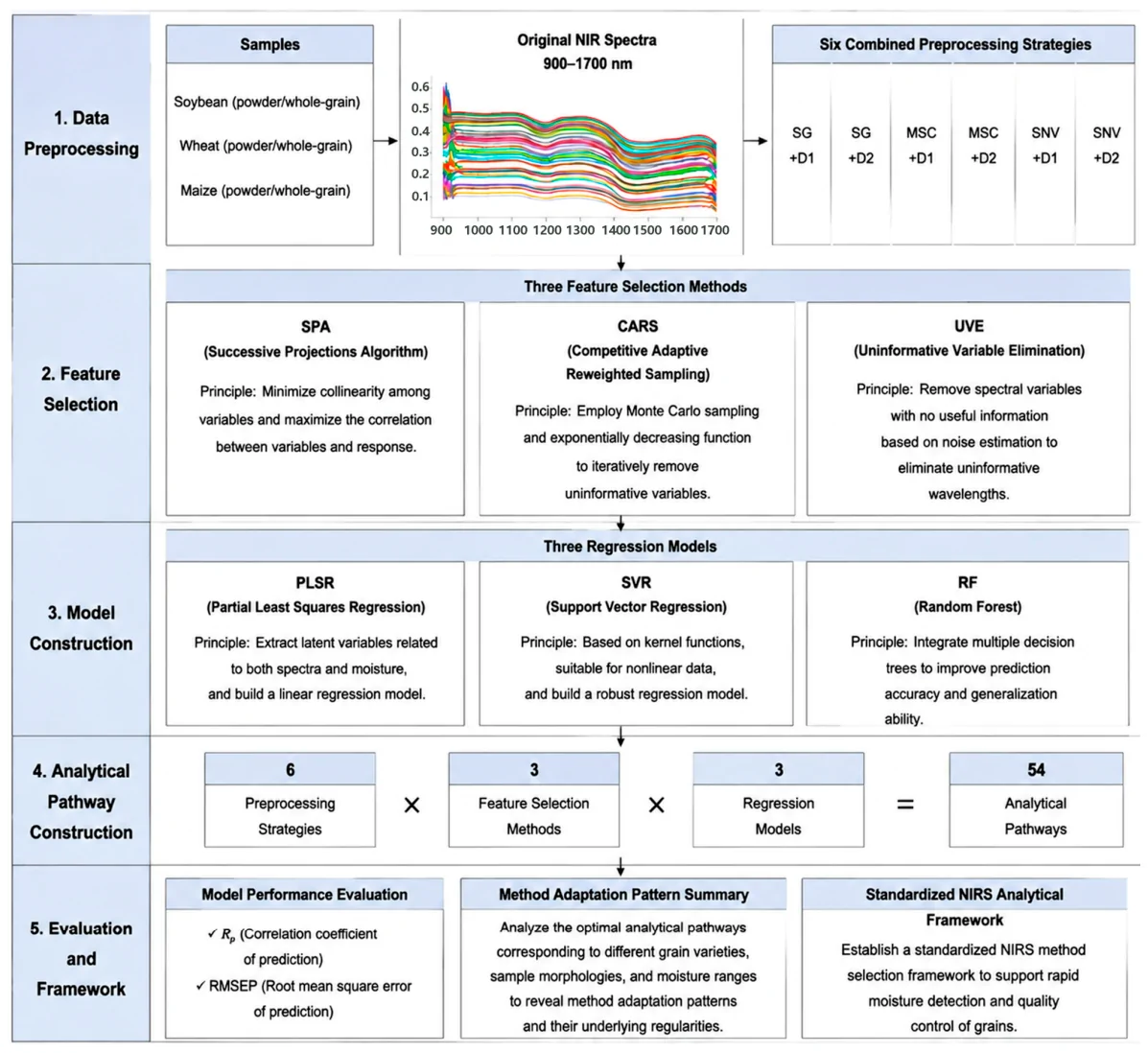
Workflow of the proposed NIRS-based method selection framework for grain moisture prediction.

The overall framework of the 54 analytical pathways and the complete analytical workflow are illustrated in Figure 2.

#### 2.4.3. Model Evaluation and Spectral Variation Analysis

The prediction correlation coefficient (Rp) was used to evaluate the linear agreement between predicted values and reference values. The calculation formula is as follows:

(3) $$R_{p}=\frac{\sum_{i=1}^{n}\left(y_{i,actual}-{\bar{y}}_{i,actual}\right)\left(y_{i,predicted}-{\bar{y}}_{i,predicted}\right)}{\sqrt{\sum_{i=1}^{n}{\left(y_{i,actual}-{\bar{y}}_{i,actual}\right)}^{2}}\times \sqrt{\sum_{i=1}^{n}{\left(y_{i,predicted}-{\bar{y}}_{i,predicted}\right)}^{2}}}$$

where n represents the number of samples in the prediction set; $y_{i,actual}$ and $y_{i,predicted}$ represent the measured and predicted values of the i sample, respectively. An Rp value closer to 1 indicates a stronger agreement between the predicted values and the reference values.

The root mean square error of prediction (RMSEP) was used to evaluate the prediction error of the model for unknown samples. The calculation formula is as follows:

(4) $$RMSEP=\sqrt{\frac{1}{n}\sum_{i=1}^{n}{\left(y_{i,actual}-{\bar{y}}_{i,predicted}\right)}^{2}}$$

where n represents the number of samples in the prediction set; $y_{i,actual}$ and ${\bar{y}}_{i,predicted}$ represent the measured and predicted values of the i sample, respectively. A lower RMSEP value indicates better prediction accuracy of the model.

In addition to model performance evaluation, spectral variation analysis was conducted to compare the spectral stability of powder and whole-grain samples. For each grain crop, 13 moisture levels were prepared. At each moisture level, spectra were collected from four measurement positions, with four separate spectral acquisitions performed at each position, resulting in 16 repeated spectral observations.

For each moisture level, the mean and standard deviation of the reflectance values from the 16 repeated spectral observations were calculated at each wavelength. The wavelength-wise coefficient of variation (CV) was then calculated according to Equation (5):

(5) $$CV\lambda =\frac{SD(R\lambda )}{\left|R\lambda \right|}\times 100\%$$

where the corresponding variables represent the mean reflectance and standard deviation of the 16 repeated spectral observations at wavelength λ under the same moisture level, respectively.

Considering that individual wavelengths may be affected by local noise and produce abnormal CV values, the median CV across the full wavelength range was used as the representative CV statistic for each moisture level. This approach reduced the influence of abnormal wavelength-specific values on the overall evaluation. Wavelengths were excluded when the absolute value of the mean reflectance was ≤0.01 or when the calculated CV value was non-finite. Finally, 13 representative CV values were obtained for each sample morphology and compared between powder and whole-grain samples.

Because the CV data did not completely satisfy the assumption of normality, a two-sided Wilcoxon signed-rank test was applied to compare powder and whole-grain samples. The matched-sample rank-biserial correlation coefficient was calculated as an effect-size indicator. In addition, 20,000 iterations of nonparametric bootstrap resampling were performed to estimate the 95% confidence intervals of the CV statistics and improve the robustness of the statistical analysis.

It should be noted that the CV analysis was performed only using repeated spectral observations obtained at the same moisture level. Its purpose was to evaluate spectral stability and spatial heterogeneity associated with different sample morphologies, and it was not involved in regression-model development, training, or performance evaluation. The CV results were used only to support interpretation of the influence of sample morphology on spectral quality and method adaptation patterns.

## 3. Results

### 3.1. Moisture Content Distribution of Samples

Thirteen moisture levels were prepared for each of the three grain crops, and the reference moisture content distributions are presented in Table 1. The moisture content of soybean samples ranged from 6.65% to 26.87%, with a mean value of 13.52% and a standard deviation of 6.03%. The moisture content ranges of wheat and maize samples were 9.38–25.72% and 9.64–29.32%, respectively, with mean values of 16.51% and 18.53% and standard deviations of 5.57% and 6.78%, respectively.

The moisture levels of all three grain crops covered a broad range from low to high moisture contents, providing a suitable moisture range for spectral acquisition and comparative model development. The mean and standard deviation values in Table 1 provide descriptive information on the central level and dispersion, respectively, of the 13 experimentally established moisture levels for each grain crop. Together with the minimum and maximum values, these statistics characterize the moisture range represented in the experimental dataset and facilitate comparison of the moisture distributions established for soybean, maize, and wheat. These moisture levels were subsequently used for spectral acquisition and model development of both whole-grain and powder samples, providing a consistent reference basis for comparing different sample morphologies and analytical pathways.

### 3.2. Original Spectral Characteristics and Influence of Sample Morphology

**Figure 3:**
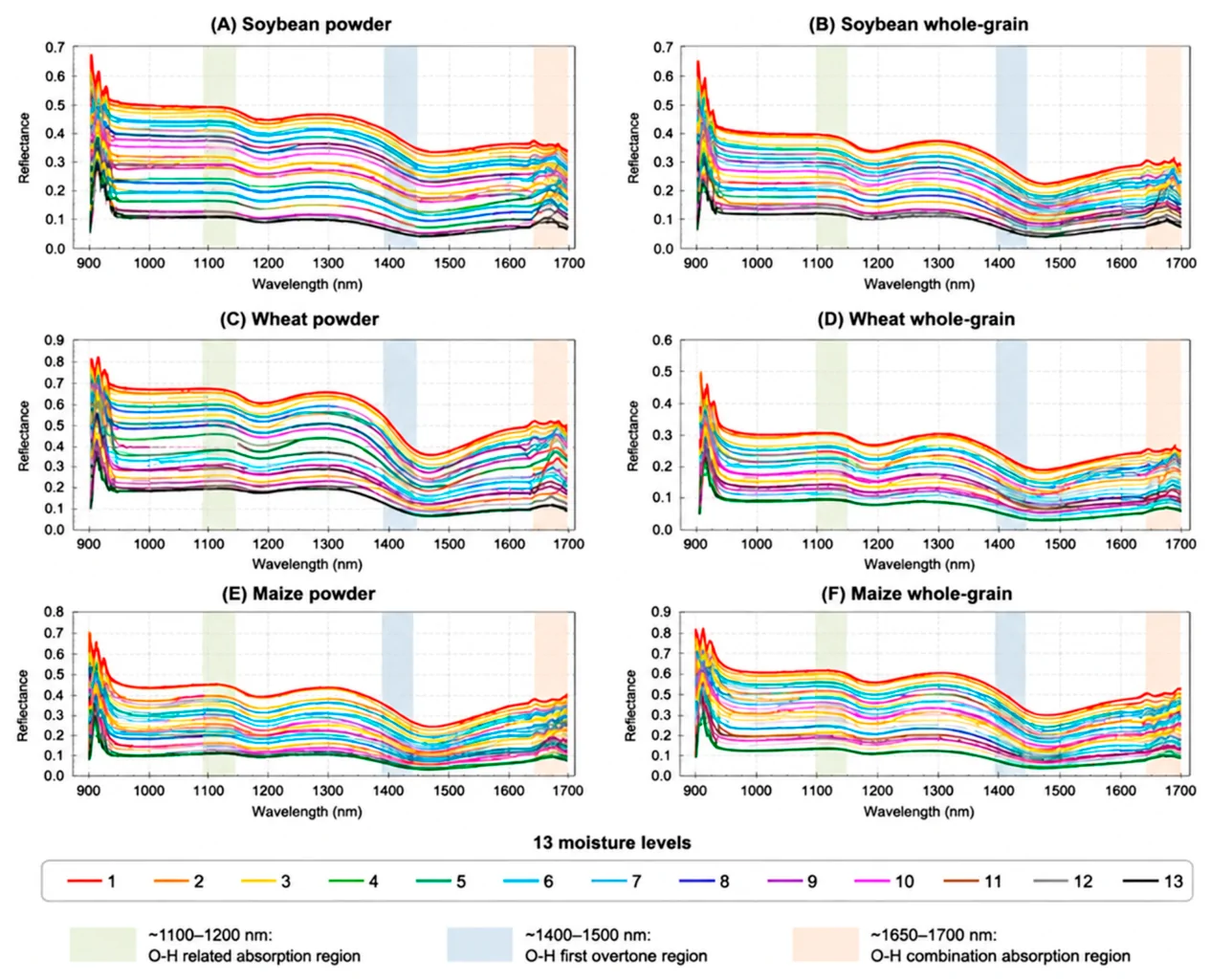
Original Spectra of Whole-Grain and Powder Samples from Three Grain Crops.

The original near-infrared spectra of whole-grain and powder samples of the three grains in the range of 900–1700 nm are shown in Figure 3. All grain species exhibited similar spectral variation trends, with obvious absorption features observed around 900–970 nm and 1400–1480 nm, which correspond to the characteristic absorption regions of O–H bonds in water. Compared with powder samples, whole-grain samples showed stronger baseline fluctuations and greater spectral differences among samples, which were mainly attributed to the heterogeneous internal structure and enhanced light scattering effects of intact grains.

After applying different spectral preprocessing strategies, the baseline drift, random noise, and scattering effects in the original spectra were reduced to different degrees. The water-related absorption features became clearer, and the overall spectral stability was improved. Among them, scattering correction methods (MSC and SNV) effectively reduced spectral variations caused by differences in sample particle state and surface structure. Meanwhile, derivative preprocessing further enhanced the spectral resolution of moisture-sensitive regions, providing more reliable spectral information for subsequent feature wavelength selection and regression modeling.

Overall, the main water-related absorption characteristics were preserved after preprocessing for different grain species and sample morphologies, while background interference was reduced. These results provided a reliable spectral basis for the subsequent comparison of analytical pathways and evaluation of their adaptation patterns.

To further evaluate the influence of sample morphology on spectral stability, the wavelength-wise coefficient of variation (CV) was calculated based on 16 repeated spectral observations collected at each moisture level. As shown in Table 2, whole-grain samples of all three grain species exhibited higher spectral variability than powder samples. The median CV values of soybean whole-grain, maize whole-grain, and wheat whole-grain were 17.43%, 29.73%, and 17.47%, respectively, which were higher than those of the corresponding powder samples (8.09%, 7.27%, and 5.95%). The spectral variability of whole-grain samples was approximately 2.2, 4.1, and 2.9 times higher than that of powder samples for soybean, maize, and wheat, respectively.

Statistical analysis showed that the differences in spectral variability between powder and whole-grain samples were significant for maize and wheat (*p* < 0.001 and *p* = 0.006), with large effect sizes (rank-biserial correlation, r_rb = 1.000 and 0.824, respectively). Although the difference for soybean samples did not reach statistical significance (*p* = 0.057), whole-grain samples still showed a tendency toward higher spectral variability. These results indicate that powder samples can effectively reduce spectral fluctuations caused by internal structural differences, thereby improving spectral repeatability and measurement stability in near-infrared analysis.

Therefore, sample morphology not only affects the stability of near-infrared spectra but also influences the suitability of feature wavelength selection and modeling methods. Powder samples generally provide more stable spectral information due to their better homogeneity, whereas whole-grain samples require appropriate preprocessing and feature selection strategies to reduce the effects of scattering and structural differences.

### 3.3. Feature Selection Results

The characteristic wavelength selection results corresponding to the optimal analytical pathways are presented in Table 3. The number of selected variables varied among different grain varieties, sample morphologies, preprocessing strategies, and feature selection algorithms. Among the six representative optimal pathways, the numbers of selected wavelengths ranged from 27 to 52. These results indicate that different feature selection methods retained different amounts of spectral information according to the characteristics of the corresponding samples and preprocessing strategies.

Different feature selection algorithms exhibited distinct variable selection characteristics. CARS was selected in four of the six representative optimal pathways, with 42, 31, 28, and 38 wavelengths retained for soybean whole-grain, maize powder, wheat whole-grain, and wheat powder samples, respectively. SPA was selected for soybean powder samples and retained 27 characteristic wavelengths, demonstrating effective dimensionality reduction. UVE was selected for maize whole-grain samples and retained 52 characteristic wavelengths.

The suitability of feature selection algorithms varied among different grain varieties and sample morphologies. CARS showed favorable adaptability for soybean whole-grain, maize powder, wheat whole-grain, and wheat powder samples, whereas UVE was more suitable for maize whole-grain samples and SPA for soybean powder samples within their respective optimal analytical pathways. Overall, the results demonstrate that the appropriate feature selection strategy depended on the specific grain variety, sample morphology, and preceding preprocessing method. Therefore, feature selection should emphasize the adaptability between analytical strategies and target samples rather than relying on a single method for all detection objects.

#### 3.3.1. CARS-Based Feature Selection Results

**Figure 4:**
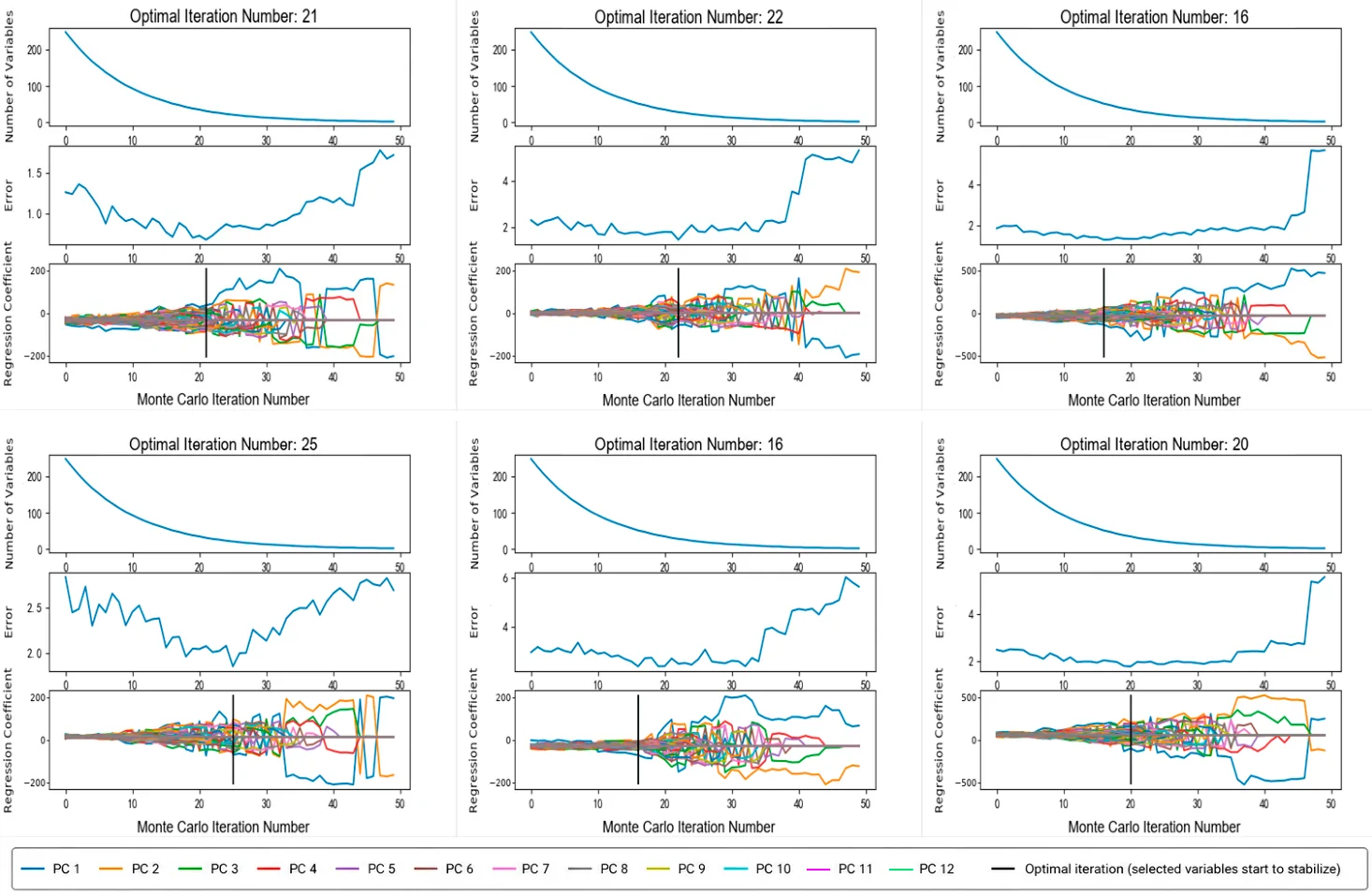
Variation Trends of Variable Number and RMSECV During CARS Algorithm Screening.

Competitive adaptive reweighted sampling (CARS) was applied to select characteristic wavelengths from the near-infrared spectra of different grain varieties and sample morphologies, and the results are shown in Figure 4. As the number of Monte Carlo iterations increased, the number of selected variables gradually decreased, while the root mean square error of cross-validation (RMSECV) initially decreased and then tended to stabilize. These results indicate that CARS can progressively eliminate redundant variables while retaining informative spectral variables closely associated with moisture content.

The optimal iteration numbers varied among different grain varieties and sample morphologies. Overall, powder samples generally required fewer iterations to achieve the minimum RMSECV compared with whole-grain samples, whereas whole-grain samples tended to require more iterations to obtain stable feature subsets. This finding suggests that sample morphology influences the feature selection process; however, the final selected characteristic wavelengths were mainly concentrated in moisture-sensitive absorption regions.

Considering the changes in variable number and RMSECV, CARS effectively removed redundant wavelengths while preserving abundant useful spectral information. The algorithm demonstrated reliable feature extraction capability across different grain varieties and provided informative spectral variables for subsequent regression model development.

#### 3.3.2. SPA-Based Feature Selection Results

**Figure 5:**
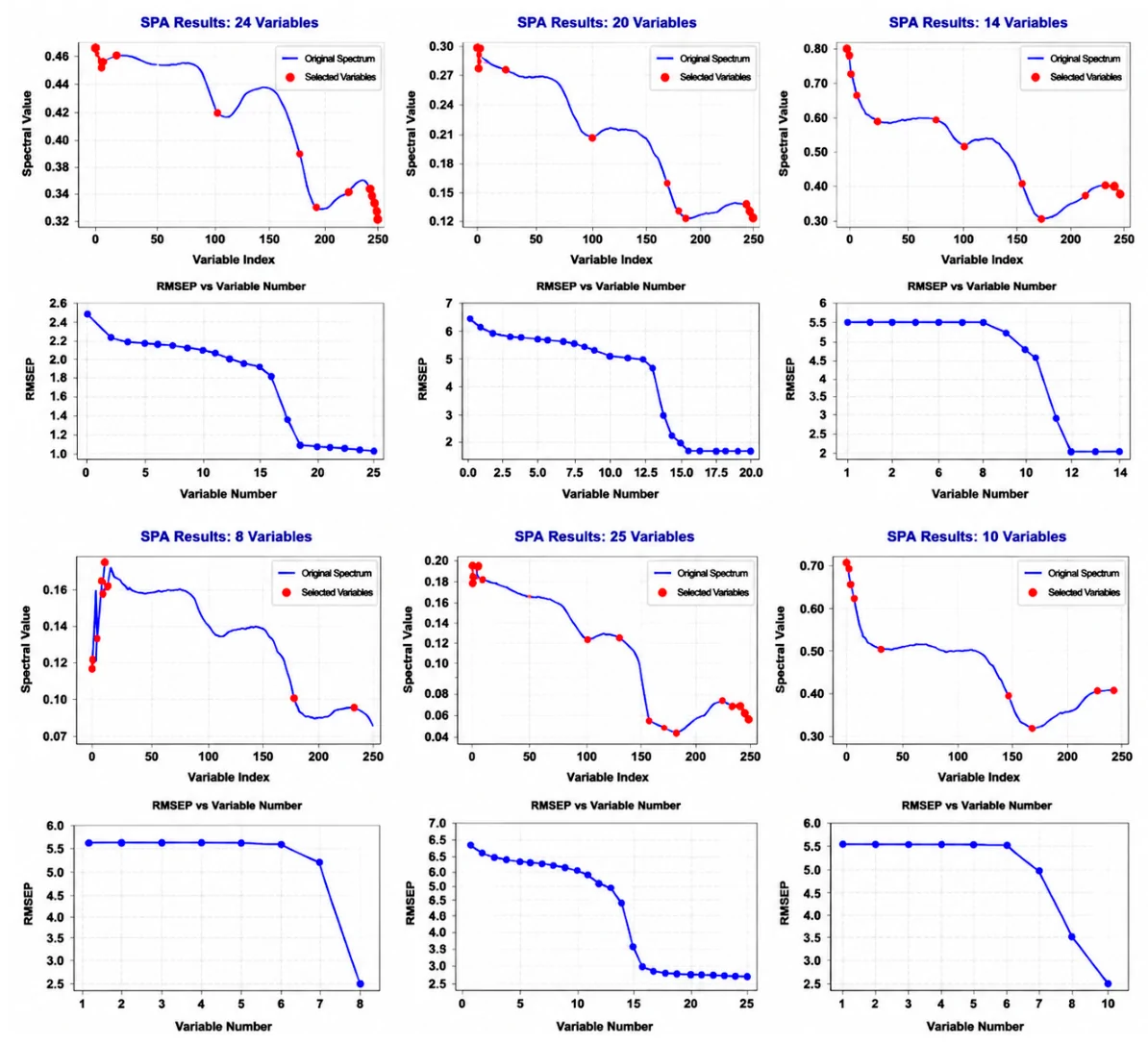
Results of Characteristic Wavelength Screening by SPA.

Successive projections algorithm (SPA) was applied to select characteristic wavelengths from the near-infrared spectra of different grain varieties and sample morphologies, and the results are shown in Figure 5. As the number of selected variables increased, the RMSEP values of all samples showed a rapid decrease followed by a gradual stabilization, indicating that SPA can effectively reduce spectral collinearity while maintaining high prediction accuracy.

The optimal numbers of selected variables varied among different grain varieties and sample morphologies. Overall, whole-grain samples generally required fewer characteristic variables than powder samples. The selected variables covered different regions of the investigated spectral range and included variables located in moisture-sensitive spectral regions, suggesting that SPA can retain relevant moisture-related spectral information with fewer variables and achieve effective dimensionality reduction.

Overall, the selection results across different grain varieties demonstrated that SPA has advantages in reducing variable redundancy and improving computational efficiency. It is particularly suitable for analytical scenarios requiring a compact set of variables and relatively simple model structures, providing representative spectral features for subsequent regression model development.

#### 3.3.3. UVE-Based Feature Selection Results

**Figure 6:**
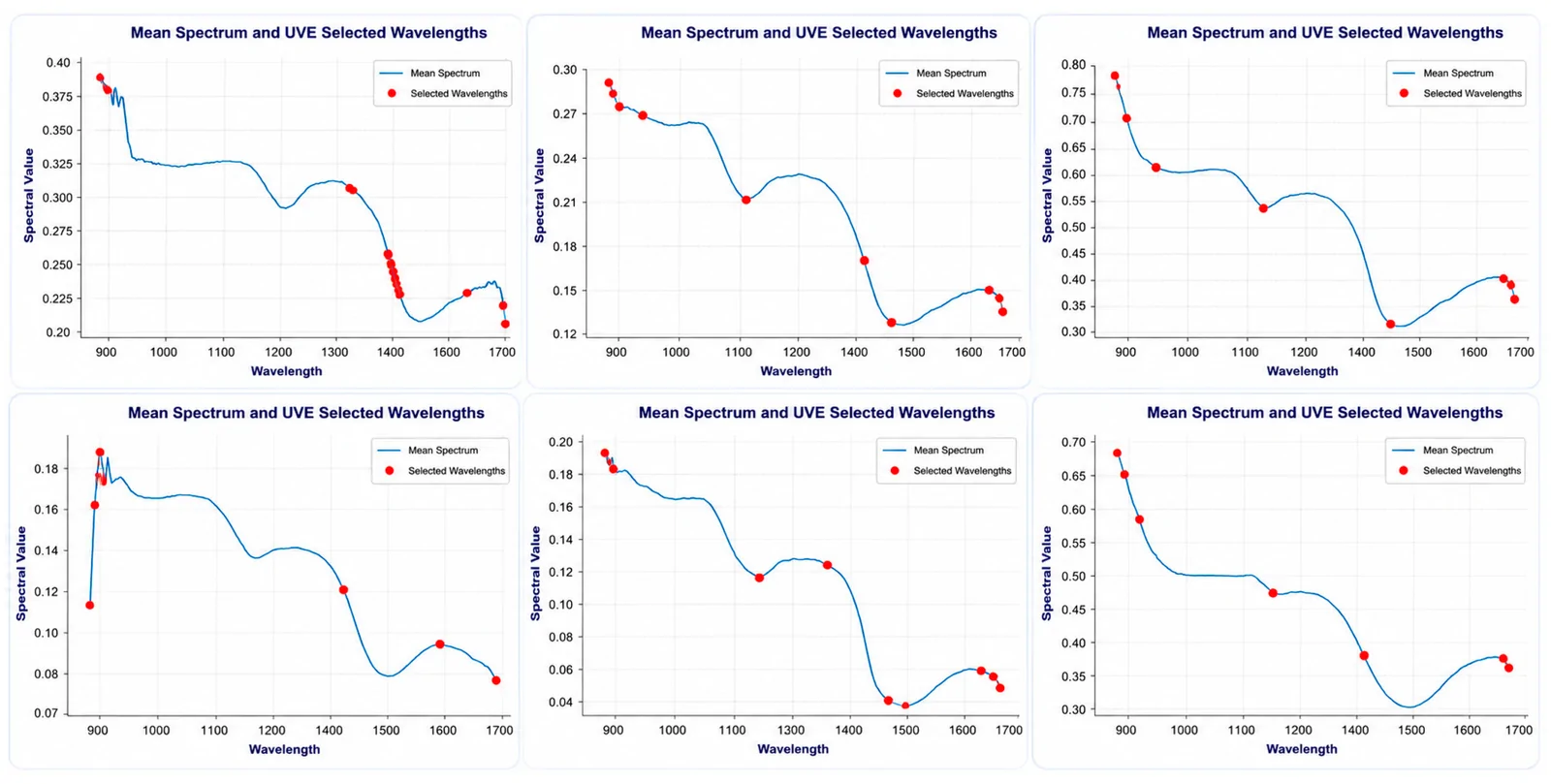
Results of UVE Characteristic Wavelength Screening for Moisture Prediction.

Uninformative variable elimination (UVE) was applied to select characteristic wavelengths from the near-infrared spectra of different grain varieties and sample morphologies, and the results are shown in Figure 6. UVE effectively removed less informative variables with limited contributions to model development while retaining relevant spectral regions closely associated with moisture prediction.

The selected characteristic wavelengths were distributed across different regions of the investigated spectral range, with several selected variables located in spectral regions associated with moisture-related absorption. This distribution indicates that UVE can effectively identify spectral information relevant to moisture prediction. Compared with full-spectrum modeling, UVE reduced the number of variables while preserving informative spectral characteristics, thereby improving the stability of subsequent prediction models.

Overall, UVE exhibited effective variable selection capability for samples with relatively strong spectral interference. It reduced the influence of redundant information on model prediction performance and provided reliable characteristic variables for developing robust moisture prediction models.

### 3.4. Model Performance Evaluation

#### 3.4.1. Evaluation of Model Performance Across 54 Analytical Pathways

**Figure 7:**
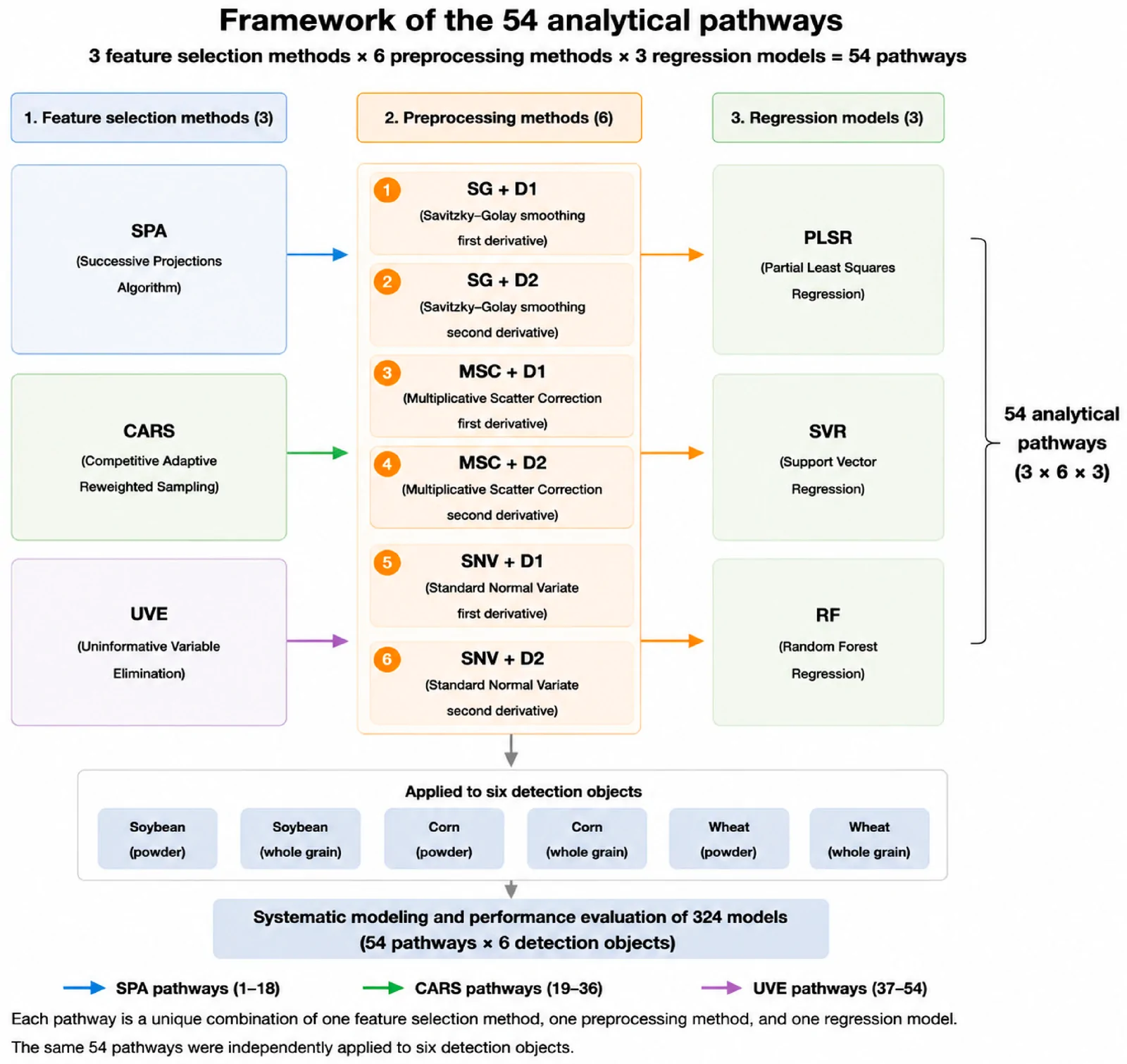
Matrix of the 54 analytical pathways constructed by combining six preprocessing strategies, three feature selection algorithms, and three regression models.

To systematically evaluate the effects of different analytical combinations on grain moisture prediction performance, six combined preprocessing strategies, three feature selection algorithms, and three regression models were fully combined to construct 54 NIRS-based analytical pathways, as illustrated in Figure 7. Each analytical pathway consisted of one preprocessing strategy, one feature selection algorithm, and one regression model. Specifically, the pathways included three feature selection algorithms (SPA, CARS, and UVE), six combined preprocessing strategies (SG + D1, SG + D2, MSC + D1, MSC + D2, SNV + D1, and SNV + D2), and three regression models (PLSR, SVR, and RF).

The 54 analytical pathways were independently applied to powder and whole-grain samples of soybean, maize, and wheat, covering six detection objects in total. Therefore, 324 independent modeling analyses and performance evaluations were conducted. All analytical pathways used the same data partition strategy and evaluation criteria. The prediction correlation coefficient (Rp) and root mean square error of prediction (RMSEP) were used as the primary indicators for model performance evaluation, ensuring consistency and fairness in comparisons among different analytical pathways.

Overall, the optimal analytical pathways varied considerably among different grain varieties and sample morphologies, and no universal optimal method combination was identified for all detection objects. Although some analytical pathways achieved the highest prediction performance, several alternative combinations showed comparable results, indicating that multiple high-performing analytical pathways may exist for a specific detection object. Therefore, representative high-performance pathways for powder and whole-grain samples of soybean, maize, and wheat are further compared in the following sections to summarize the method adaptation patterns among preprocessing strategies, feature selection algorithms, and regression models.

#### 3.4.2. Suitable Analytical Pathways for Soybean Samples

Among the 54 analytical pathways established in this study, both soybean powder and whole-grain samples achieved high moisture prediction accuracy; however, the optimal analytical pathways differed considerably between the two sample morphologies (Table 4).

For powder samples, the combination of MSC + D1 preprocessing, SPA feature selection, and PLSR modeling achieved the highest prediction performance, with an Rp of 0.9911 and an RMSEP of 0.3663%. The SNV + D1 + SPA + PLSR pathway showed comparable performance, with an Rp of 0.9879 and an RMSEP of 0.5003%, indicating that both pathways provided highly accurate moisture prediction.

For whole-grain samples, the SNV + D2 + CARS + SVR pathway achieved the best prediction performance, with an Rp of 0.9858 and an RMSEP of 0.7341%, followed by the MSC + D2 + UVE + SVR pathway (Rp = 0.9723, RMSEP = 1.4282%). Compared with powder samples, whole-grain samples exhibited slightly higher prediction errors; however, the results still demonstrated favorable prediction performance for whole-grain moisture assessment under the present experimental conditions.

Overall, PLSR-based pathways showed better performance for soybean powder samples, whereas SVR-based pathways achieved higher prediction accuracy for whole-grain samples. The optimal analytical pathways varied between the two sample morphologies, and the difference between the best and second-best pathways was relatively small for powder samples. These results indicate that soybean samples may have multiple suitable analytical pathways with high prediction capability rather than a single optimal solution.

#### 3.4.3. Suitable Analytical Pathways for Maize Samples

Among all analytical pathways, both maize powder and whole-grain samples achieved high prediction accuracy (Table 5), and the random forest (RF) model generally exhibited good prediction stability.

For powder samples, the MSC + D1 + CARS + RF analytical pathway achieved the highest prediction performance, with an Rp of 0.9865 and an RMSEP of 0.5784%. The MSC + D1 + UVE + RF pathway showed comparable performance, with an Rp of 0.9830 and an RMSEP of 0.7312%.

For whole-grain samples, the MSC + D1 + UVE + RF pathway achieved the best prediction performance, with an Rp of 0.9833 and an RMSEP of 0.6599%. The SNV + D1 + CARS + RF pathway showed similar performance, with an Rp of 0.9826 and an RMSEP of 0.6877%.

Overall, the RF model exhibited good prediction capability for both maize powder and whole-grain samples, while different feature selection algorithms showed different suitability depending on sample morphology. Since the differences between the best-performing and second-best pathways were relatively small, maize samples, similar to soybean samples, may also have multiple suitable analytical pathways with high prediction capability.

#### 3.4.4. Suitable Analytical Pathways for Wheat Samples

Among all analytical pathways, wheat samples showed slightly lower overall prediction accuracy compared with soybean and maize samples; however, differences among analytical pathways were still observed (Table 6).

For powder samples, the SNV + D2 + CARS + SVR analytical pathway achieved the highest prediction performance, with an Rp of 0.9730 and an RMSEP of 0.8767%. The MSC + D1 + SPA + PLSR pathway showed the second-best performance, with an Rp of 0.9604 and an RMSEP of 1.2850%.

For whole-grain samples, the MSC + D1 + CARS + SVR pathway achieved the highest prediction performance, with an Rp of 0.9587 and an RMSEP of 1.1041%. The SNV + D1 + CARS + SVR pathway showed comparable performance, with an Rp of 0.9525 and an RMSEP of 1.2714%.

Overall, wheat powder samples exhibited higher prediction accuracy than whole-grain samples, and the SVR model showed good applicability for both sample morphologies. Although the prediction performance varied among different analytical pathways, multiple pathways achieved relatively high prediction accuracy, indicating that wheat samples also possess several suitable analytical strategies rather than a single optimal analytical pathway.

#### 3.4.5. Suitable Analytical Pathways and Method Adaptation Patterns

Based on the combination of six preprocessing strategies, three feature selection algorithms, and three regression models, a total of 54 NIRS-based analytical pathways were established in this study. The prediction performances of different analytical combinations were systematically compared across three grain crops and two sample morphologies. The results demonstrated that the high-performing analytical pathways varied among different detection objects. Therefore, the main objective of this study was not to identify a single optimal model applicable to all detection objects, but rather to reveal the method adaptation patterns among different grain varieties and sample morphologies.

To further summarize the representative high-performance analytical pathways for different detection objects and analyze the applicability characteristics of different analytical components, the preprocessing strategies, feature selection algorithms, and regression models corresponding to the best-performing pathways among the six detection objects were statistically analyzed. The results are presented in Table 7.

According to Table 7, MSC + D1 was the most frequently selected preprocessing strategy, appearing in four out of the six analytical objects (66.7%). CARS was the most frequently applied feature selection algorithm, also occurring four times (66.7%). Among the regression models, SVR showed the highest occurrence frequency, appearing in three cases (50.0%), followed by RF (two cases) and PLSR (one case). Although MSC + D1, CARS, and SVR exhibited relatively high applicability, SNV + D2, SPA, UVE, RF, and PLSR also showed advantages for specific analytical objects. These results indicate that different analytical methods have different application ranges, and no single analytical pathway can be universally applied to all grain varieties and sample morphologies. Therefore, the results further confirm the existence of distinct method adaptation patterns in NIRS-based grain moisture prediction.

**Figure 8:**
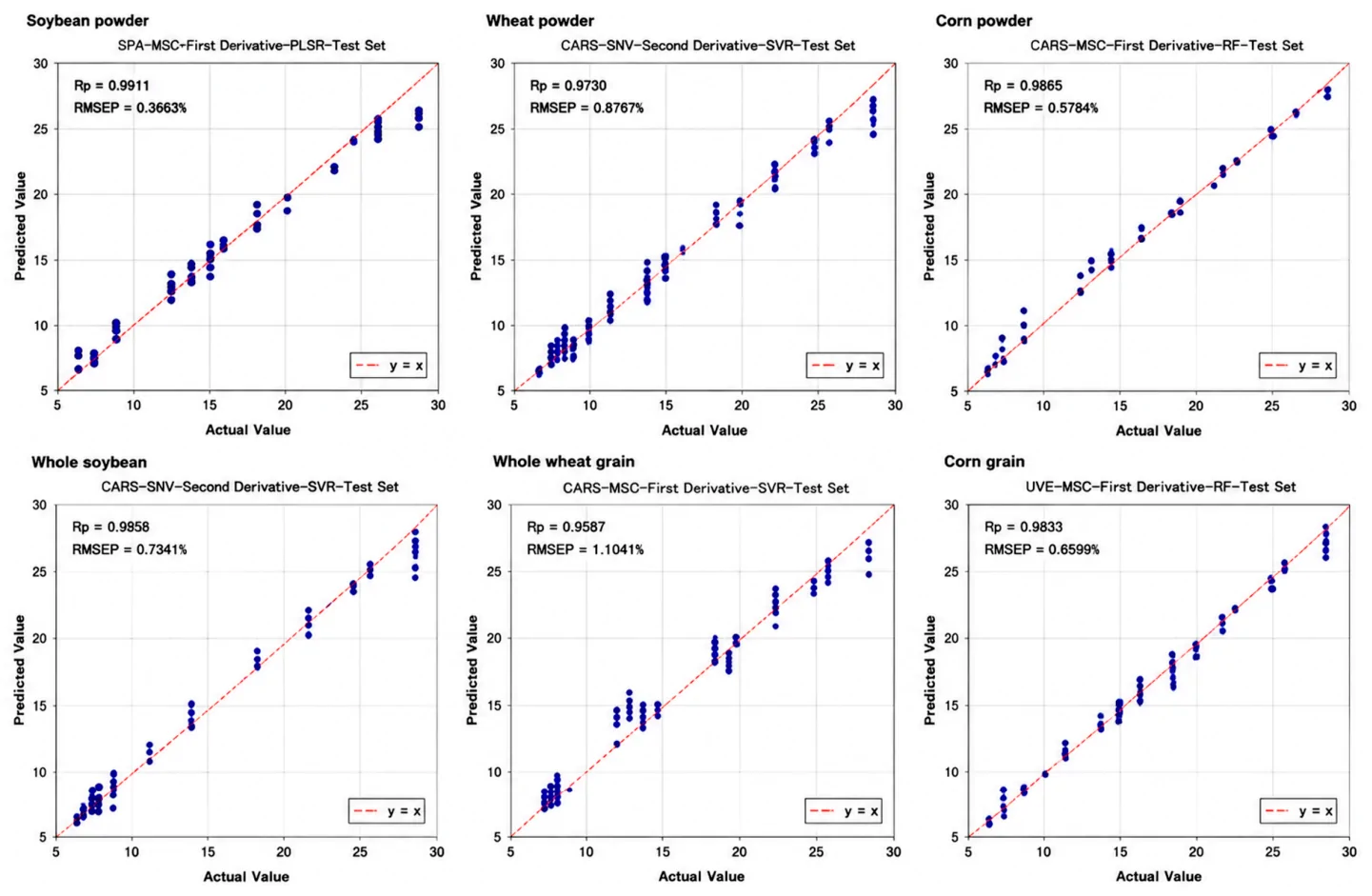
Comparison of measured and predicted values of representative analytical pathways for three grains. Blue dots represent individual prediction samples.

Figure 8 presents the prediction results of six representative analytical pathways. The predicted values showed strong linear relationships with the measured values, and most scatter points were distributed close to the 1:1 reference line, indicating that the developed models effectively captured the quantitative relationship between spectral information and moisture content. The representative high-performance pathways for soybean powder, wheat powder, and maize powder samples were MSC + D1-SPA-PLSR, SNV + D2-CARS-SVR, and MSC + D1-CARS-RF, respectively. For whole-grain samples, the corresponding pathways were SNV + D2-CARS-SVR, MSC + D1-CARS-SVR, and MSC + D1-UVE-RF, respectively.

Further comparison between powder and whole-grain samples revealed that powder samples generally achieved higher prediction accuracy and lower prediction errors. Although whole-grain samples were affected by differences in grain structure and optical path length, stable prediction performance could still be obtained through appropriate combinations of spectral preprocessing, feature selection algorithms, and regression models. Therefore, different grain varieties and sample morphologies did not exhibit an identical optimal analytical workflow but instead showed distinct method adaptation patterns.

**Figure 9:**
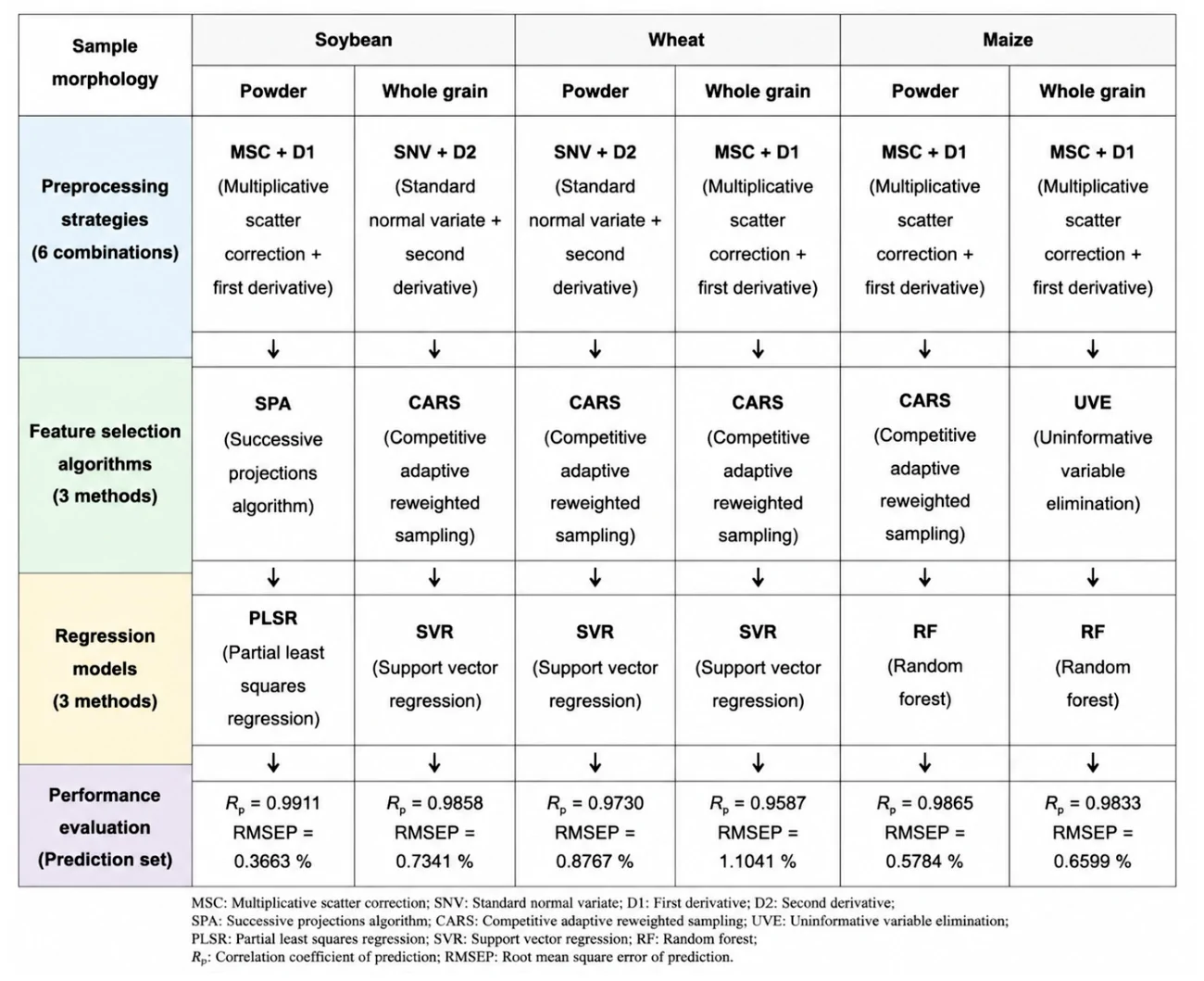
Method adaptation patterns and NIRS-based method selection framework for moisture prediction of soybean, maize and wheat.

Based on the above findings, a NIRS-based method selection framework for moisture prediction of soybean, wheat, and maize was further proposed in this study (Figure 9). The framework starts from grain variety and sample morphology and enables the selection of suitable analytical pathways by matching spectral preprocessing strategies, feature selection algorithms, and regression models. It establishes an analytical logic consisting of “grain variety–sample morphology–spectral preprocessing–feature selection–regression modeling–suitable analytical pathway”. This framework aims to provide guidance for the rational selection of NIRS analytical methods under different detection scenarios rather than replacing external validation or inter-instrument transfer validation.

## 4. Discussion

### 4.1. Method Adaptation Patterns and Mechanistic Interpretation

The present study demonstrated that the prediction performance of NIRS-based moisture models was not determined by a single analytical method, but rather by the synergistic optimization of spectral preprocessing, feature selection, and regression modeling. Spectral preprocessing reduces scattering effects, baseline drift, and noise interference; feature selection extracts informative variables associated with moisture content; and regression models establish quantitative relationships between spectral characteristics and moisture content. Therefore, the major contribution of this study was not to identify a single optimal model applicable to all analytical objects, but to reveal the method adaptation patterns among different grain varieties and sample morphologies through a systematic comparison of 54 analytical pathways.

Previous studies have demonstrated the potential of NIRS for grain quality assessment. Zhu et al. [27], Zou et al. [28], Xu et al. [29], and Xiang et al. [30] verified the applicability of NIRS in grain quality evaluation, characteristic wavelength selection, and rapid moisture prediction. However, most previous studies focused on a single grain variety or a specific analytical workflow, and systematic comparisons among different grain varieties and sample morphologies remain limited. In this study, an integrated evaluation system was established under unified experimental conditions, covering three major grain crops (soybean, maize, and wheat) and two sample morphologies (whole-grain and powder samples). This provides a reference for selecting appropriate NIRS analytical strategies for different analytical objects.

The experimental results showed that powder samples generally exhibited better prediction performance than whole-grain samples. This difference can be primarily interpreted from the physical characteristics of NIR measurement and sample morphology. For intact grains, the NIR signal represents only the optically sampled region of the kernel, while differences in kernel geometry, surface structure, internal composition, and measurement position can introduce additional variation in light scattering and optical path length. Consequently, the measured spectrum may not fully represent the spatially heterogeneous moisture distribution within the whole kernel. The limited penetration depth of NIR radiation further restricts the effective sampling volume and can increase the influence of local heterogeneity on the measured spectral response [31]. Grinding disrupts the intact kernel structure and produces a more homogeneous sample matrix, thereby reducing spatial heterogeneity and variations in light scattering and improving the representativeness and repeatability of spectral measurements. This interpretation is consistent with the CV analysis in the present study, in which whole-grain samples of all three crops exhibited higher spectral variability than their corresponding powder samples. Nevertheless, whole-grain samples still achieved satisfactory prediction performance through appropriate analytical pathway selection, supporting their potential for rapid nondestructive moisture assessment.

Different grain varieties exhibited different high-performance analytical pathways, which may be associated not only with differences in kernel structure but also with differences in their chemical matrices. Soybean, maize, and wheat differ substantially in the relative proportions of major constituents such as starch, protein, and lipids. These compositional differences can influence water adsorption and retention because hydrophilic surfaces associated with starches, proteins, and other grain constituents contribute differently to water binding [32]. Such differences may consequently affect moisture-related spectral responses, as NIR spectra contain overlapping absorption information associated with O–H, C–H, and N–H bonds. Therefore, the spectral response to moisture is embedded within different chemical backgrounds for the three grain crops, which may partly explain why identical preprocessing, feature-selection, and regression strategies did not provide uniformly optimal performance across all analytical objects. Together with differences in kernel structure and sample morphology, these matrix effects support the observed grain-dependent method adaptation patterns. Therefore, selecting suitable analytical pathways according to the specific detection object may be more appropriate than assuming that a single analytical workflow is universally optimal.

Overall, this study revealed the adaptation relationships among grain varieties, sample morphologies, and analytical strategies. The spectral variation analysis further provided physical evidence for the influence of sample structure on model performance, supporting the establishment of a NIRS-based method selection framework for different grain moisture prediction scenarios.

### 4.2. Comparison with Previous Studies and Statistical Interpretation

Previous studies on NIRS-based grain analysis have mainly focused on a single grain variety, a specific sample morphology, or optimization of a particular modeling approach. Although these studies have improved model performance, they provide limited insight into the applicability of analytical methods across different detection objects.

In this study, soybean, maize, and wheat, as well as powder and whole-grain sample morphologies, were simultaneously investigated under unified experimental conditions. A total of 54 analytical pathways were systematically compared. The results demonstrated that all grain varieties achieved high prediction performance, and powder samples generally showed better prediction accuracy than whole-grain samples, indicating the reliability of the proposed NIRS-based method selection framework.

It is noteworthy that, for most analytical objects, the differences between the best-performing and second-best pathways were relatively small. This finding suggests that NIRS-based moisture prediction does not rely on a single optimal analytical workflow applicable to all grain varieties and sample morphologies, but rather involves multiple suitable analytical pathways with high prediction capability. Therefore, the major contribution of this study lies not only in achieving high prediction accuracy but also in revealing the method adaptation patterns between analytical objects and modeling strategies, providing a more rational basis for analytical pathway selection under different application scenarios.

### 4.3. Method Selection Strategies for Different Application Scenarios

For NIRS-based grain moisture detection, prediction accuracy alone is insufficient; detection efficiency and practical application requirements should also be considered.

For laboratory analysis and quality control during grain processing, powder samples can be preferentially selected and combined with suitable analytical pathways for different grain varieties to achieve higher prediction accuracy.

For grain purchasing, storage management, and potential rapid field detection, whole-grain samples offer the advantage of nondestructive measurement without additional grinding. Although their prediction performance was slightly lower than that of powder samples under the present experimental conditions, the results demonstrate the potential of whole-grain NIRS for rapid moisture assessment. However, its performance under practical field conditions requires further validation using independent samples from different sources, batches, and environmental conditions.

Overall, the proposed method selection strategy is based on the identified method adaptation patterns and provides guidance for selecting appropriate NIRS analytical workflows under different detection scenarios. The proposed framework is intended as a methodological guidance framework rather than an automated decision-making system. Its primary purpose is to provide references for selecting suitable analytical pathways according to grain variety and sample morphology based on systematic comparative analysis.

### 4.4. Limitations and Future Perspectives

Although this study systematically compared 54 analytical pathways and proposed a NIRS-based method selection framework for different grain varieties and sample morphologies, several limitations remain.

Although the proposed framework showed stable performance under the established experimental conditions, its generalization capability under practical grain production and storage scenarios requires further validation. In this study, moisture gradients were artificially established by adding distilled water to dried grain samples, followed by sufficient equilibration, which enabled the construction of a wide and controlled moisture range for systematic comparison among analytical pathways. However, the moisture distribution state obtained through artificial conditioning may differ from that of naturally harvested or stored grains due to differences in water migration, binding state, and internal moisture distribution. In addition, the current validation was mainly conducted under laboratory conditions, and further evaluation using independent samples from different geographic origins, production batches, harvest years, and natural drying or storage processes is necessary to assess the robustness and practical applicability of the proposed framework.

First, this study established 13 moisture levels for each grain variety, and the experimental samples were mainly designed to cover the target moisture ranges. Future studies should include samples from more diverse sources, production batches, and varieties for external validation, thereby further evaluating the stability and generalization capability of the identified method adaptation patterns.

Second, sample preparation and spectral measurements were conducted under laboratory conditions, with the ambient temperature ranging approximately from 23 to 26 °C and the relative humidity from 45% to 60% during the experimental period. Although the samples were sealed immediately after preparation to minimize moisture exchange with the surrounding environment, residual moisture exchange, particularly for powder samples, cannot be completely excluded. Future studies should further evaluate the influence of environmental temperature and relative humidity on spectral stability and model performance under more strictly controlled and practical measurement conditions.

Third, the present study was conducted using laboratory-based NIRS equipment. Further investigations involving cross-batch validation, portable instrument validation, and model transfer strategies are required to improve the applicability of the proposed framework under practical detection conditions.

In addition, this study mainly focused on moisture content prediction. Future research could extend the proposed framework to other quality indicators, such as protein, fat, and starch content, and integrate model transfer, multi-source information fusion, and intelligent modeling approaches to further explore the applicability of NIRS in rapid grain quality assessment and online monitoring.

Overall, this study revealed the method adaptation patterns among different grain varieties and sample morphologies and proposed a NIRS-based method selection framework for moisture prediction of major grain crops. The findings provide a methodological reference for selecting appropriate analytical strategies under the experimental conditions investigated in this study.

## 5. Conclusions

This study investigated three major grain crops, including soybean, maize, and wheat, and systematically compared 54 NIRS-based analytical pathways constructed from six spectral preprocessing strategies, three feature selection algorithms, and three regression models. A unified evaluation framework was established to assess the applicability of different analytical strategies. The results demonstrated that powder samples generally achieved better prediction performance than whole-grain samples. The representative high-performance pathways for soybean, maize, and wheat powder samples achieved prediction correlation coefficients (Rp) of 0.9911, 0.9865, and 0.9730, respectively, while all whole-grain samples achieved Rp values of ≥0.9587. These results indicate that the proposed analytical framework enables reliable moisture prediction across different grain varieties.

Further analysis revealed that high-performance analytical pathways varied among different grain varieties and sample morphologies. Moreover, the differences between several high-performing pathways were relatively small for some analytical objects, suggesting that NIRS-based grain moisture prediction does not depend on a single universal model applicable to all objects, but instead exhibits distinct method adaptation patterns. In addition, spectral variation analysis demonstrated that whole-grain samples exhibited higher spectral variability, indicating that sample morphology is an important factor affecting model adaptability.

Based on these findings, this study proposed a NIRS-based method selection framework for moisture prediction of major grain crops. Under the experimental conditions of this study, the framework provides a systematic reference for selecting appropriate NIRS analytical workflows according to grain variety and sample morphology. Its applicability to practical grain storage, processing, and field detection requires further validation using independent samples from diverse sources, varieties, batches, and environmental conditions. Future studies should also investigate portable NIRS instrument validation, model transfer, and cross-instrument calibration to further evaluate and improve the generalization capability of the proposed framework.

## Figures and Tables

**Table 1:** Designed moisture gradients of soybean, maize and wheat samples.

Gradient Number	Soybean Moisture Content (%)	Wheat Moisture Content (%)	Maize Moisture Content (%)
1	6.65	9.38	9.64
2	7.31	9.59	9.92
3	7.38	9.80	12.75
4	7.54	12.39	13.84
5	8.15	13.05	14.95
6	8.56	13.68	16.82
7	9.32	14.49	17.55
8	11.56	17.68	18.67
9	13.94	18.10	20.60
10	18.62	21.66	22.33
11	21.55	23.81	25.68
12	24.29	25.70	29.21
13	26.87	25.72	29.32
Statistical standard			
Range (%)	6.65~26.87	9.38~25.72	9.64~29.32
Mean (%)	13.52	16.51	18.53
Standard deviation (%)	6.03	5.57	6.78

**Table 2:** Comparison of spectral variability between powder and whole-grain samples based on median wavelength-wise coefficient of variation (CV).

Grain Species	Powder CV(%)	Whole-Grain CV(%)	Wilcoxon *p*-Value	Rank-Biserial Correlation (r_rb)
Soybean	8.09	17.43	0.057	0.604
Maize	7.27	29.73	<0.001	1.000
Wheat	5.95	17.47	0.006	0.824

**Table 3:** Feature Selection Results Corresponding to the Optimal Analytical Pathways.

Sample Morphology	Optimal Preprocessing	Feature Selection Algorithm	Number of Selected Wavelengths
Soybean whole-grain	SNV + D2	CARS	42
Soybean powder	MSC + D1	SPA	27
Maize whole-grain	MSC + D1	UVE	52
Maize powder	MSC + D1	CARS	31
Wheat whole-grain	MSC + D1	CARS	28
Wheat powder	SNV + D2	CARS	38

**Table 4:** Comparison of Model Performance Using Different Preprocessing and Feature Selection Combinations for Soybean Powder and Whole-Grain Samples.

Sample Morphology	Preprocessing Method	Feature Selection Algorithm	Modeling	Prediction Performance (Rp/RMSEP%)
Powder	MSC + D1	SPA	PLSR	Rp = 0.9911/RMSEP = 0.3663
Powder	SNV + D1	SPA	PLSR	Rp = 0.9879/RMSEP = 0.5003
Whole-grain	SNV + D2	CARS	SVR	Rp = 0.9858/RMSEP = 0.7341
Whole-grain	MSC + D2	UVE	SVR	Rp = 0.9723/RMSEP = 1.4282

**Table 5:** Comparison of Model Performance Using Different Preprocessing and Feature Selection Combinations for Maize Powder and Whole-Grain Samples.

Sample Morphology	Preprocessing Method	Feature Selection Algorithm	Modeling	Prediction Performance (Rp/RMSEP%)
Powder	MSC + D1	CARS	RF	Rp = 0.9865/RMSEP = 0.5784
Powder	MSC + D1	UVE	RF	Rp = 0.9830/RMSEP = 0.7312
Whole-grain	MSC + D1	UVE	RF	Rp = 0.9833/RMSEP = 0.6599
Whole-grain	SNV + D1	CARS	RF	Rp = 0.9826/RMSEP = 0.6877

**Table 6:** Comparison of Model Performance Using Different Preprocessing and Feature Selection Combinations for Wheat Powder and Whole-Grain Samples.

Sample Morphology	Preprocessing Method	Feature Selection Algorithm	Modeling	Prediction Performance (Rp/RMSEP%)
Powder	SNV + D2	CARS	SVR	Rp = 0.9730/RMSEP = 0.8767
Powder	MSC + D1	SPA	PLSR	Rp = 0.9604/RMSEP = 1.2850
Whole-grain	MSC + D1	CARS	SVR	Rp = 0.9587/RMSEP = 1.1041
Whole-grain	SNV + D1	CARS	SVR	Rp = 0.9525/RMSEP = 1.2714

**Table 7:** Statistical summary of the advantageous analytical components underlying the Method Adaptation Pattern.

Analytical Component	Method	Frequency	Percentage
Preprocessing	MSC + D1	4	66.7%
	SNV + D2	2	33.3%
Feature selection	CARS	4	66.7%
	SPA	1	16.7%
	UVE	1	16.7%
Regression model	SVR	3	50.0%
	RF	2	33.3%
	PLSR	1	16.7%

## Data Availability

The original contributions presented in this study are included in the article. Further inquiries can be directed to the corresponding author.
